# Sensitive operation of enzyme-based biodevices by advanced signal processing

**DOI:** 10.1371/journal.pone.0198913

**Published:** 2018-06-18

**Authors:** Stanislav Mazurenko, Sarka Bidmanova, Marketa Kotlanova, Jiri Damborsky, Zbynek Prokop

**Affiliations:** 1 Loschmidt Laboratories, Department of Experimental Biology and Research Centre for Toxic Compounds in the Environment RECETOX, Masaryk University, Brno, Czech Republic; 2 International Clinical Research Center, St. Anne's University Hospital, Brno, Czech Republic; 3 Enantis, s r.o., Brno, Czech Republic; Universidade Nova de Lisboa, PORTUGAL

## Abstract

Analytical devices that combine sensitive biological component with a physicochemical detector hold a great potential for various applications, e.g., environmental monitoring, food analysis or medical diagnostics. Continuous efforts to develop inexpensive sensitive biodevices for detecting target substances typically focus on the design of biorecognition elements and their physical implementation, while the methods for processing signals generated by such devices have received far less attention. Here, we present fundamental considerations related to signal processing in biosensor design and investigate how undemanding signal treatment facilitates calibration and operation of enzyme-based biodevices. Our signal treatment approach was thoroughly validated with two model systems: (i) a biodevice for detecting chemical warfare agents and environmental pollutants based on the activity of haloalkane dehalogenase, with the sensitive range for bis(2-chloroethyl) ether of 0.01–0.8 mM and (ii) a biodevice for detecting hazardous pesticides based on the activity of γ-hexachlorocyclohexane dehydrochlorinase with the sensitive range for γ-hexachlorocyclohexane of 0.01–0.3 mM. We demonstrate that the advanced signal processing based on curve fitting enables precise quantification of parameters important for sensitive operation of enzyme-based biodevices, including: (i) automated exclusion of signal regions with substantial noise, (ii) derivation of calibration curves with significantly reduced error, (iii) shortening of the detection time, and (iv) reliable extrapolation of the signal to the initial conditions. The presented simple signal curve fitting supports rational design of optimal system setup by explicit and flexible quantification of its properties and will find a broad use in the development of sensitive and robust biodevices.

## Introduction

There is great interest in the development of simple bioanalytical systems because of their high sensitivity, specificity, rapid responses, compatibility with on-line and on-site control configurations, and cost-effectiveness. Because of these qualities, they are potentially attractive tools for detecting metabolites, hazardous chemicals, toxins or pathogens in clinical, environmental, food, and biological warfare analyses [1, 2]. Advances in enzyme-based biosensors for hazardous chemicals have been published recently [3, 4, 5]. The hardware and systems used to generate signals in such devices have been studied extensively [6, 7]. While conventional laboratory techniques, such as gas chromatography (GC) and mass spectrometry, usually demonstrate higher sensitivity and selectivity, they require sophisticated and expensive instrumentation, highly skilled staff, and usually time-consuming sample transportation and pretreatment. In contrast, portable biosensors are usually cheap, simple to use, and able to measure pollutants in complex matrices with minimal sample pretreatment [8]. Moreover, they provide fast response time, continuous signal enabling a real-time monitoring data, the possibility of miniaturization, and portability, which permits their use as field devices working on-site.

Among a variety of methods in biosensing, electrochemical and optical techniques are most widely used in the development of catalytic biosensors [9, 10]. Potentiometric systems often require only simple and inexpensive instrumentation and exhibit a wide detection range, high sensitivity, and selectivity due to the ion-selective working electrode. However, a highly stable and accurate reference electrode is required. A wide range of potential problems coming from complex environmental samples, namely electrode fouling, drifting, dissolution, and general instability may result in erroneous response characteristics, poor reproducibility, and a high surface excess of analyte that degrades the detection limit of the ion-selective electrode [11]. Optical biosensors measure optical signal changes, e.g., due to the changing concentration of protons as a result of an enzymatic reaction, and offer similar benefits such as miniaturization and cost-effectiveness [12]. Moreover, these biosensors do not require additional reference element, but tend to have a narrower dynamic range compared to the electrodes, higher sensitivity to the sample turbidity, the ambient/scattered excitation light, and the dye quality [13].

Irrespective of the type of biorecognition element, the output signal is usually time-dependent. However, there have been few comprehensive studies on the mathematical analysis of the signal generated by the biosensor even though signal processing is at least as important for effective use of biodevices as the development of a robust biochemical system. Most systematic research on the mathematical modeling and analytical treatment of biodevice signals has focused on devices for enzyme characterization and interaction analysis [14, 15]; existing substance detectors primarily use *ad-hoc* data treatments, and only a few publications described more systematic approaches based on mathematical modeling [16]. Some notable exceptions include recent publications describing the use of the Gaussian Process for efficient calibration of sensors in drifting environments [17], reservoir computing algorithms to overcome the slow temporal dynamics of metal oxide gas sensor arrays [18], a system of reaction-diffusion equations for the detection of Hg(II) with an inhibition-based biosensor [19], a mathematical model for a single-enzyme two-substrate biocatalytic amperometric biosensor [20], a two-substrate mathematical model of microspherical optical enzymatic glucose sensors [21], and a study on validity of one particular type of functions for describing calibration curves [22].

While these contributions are important, there is little literature discussing and systemizing signal processing techniques for biosensors. This paper aims to fill the gap by comparing and contrasting simple methods with those employing mathematical modeling. The commonly used standard procedure for performing analyte concentration measurements is to monitor the response of a biodevice to an analyte concentration at a fixed time after mixing and then plotting the amplitude (i.e., the difference in signal intensity before and after mixing) or slope of the response against concentration. Thus, a significant part of the information is not used because only two points or an initial fraction of the signal curve are analyzed. In contrast, modeling of signal curves usually involves curve-fitting and thus uses the whole signal curve, but challenges the researchers with additional tools for data analysis. The problem of the choice between these two approaches is bound to arise in every development of biosensors as long as precision, robustness, and automation of signal collection are considered.

**Fig 1:**
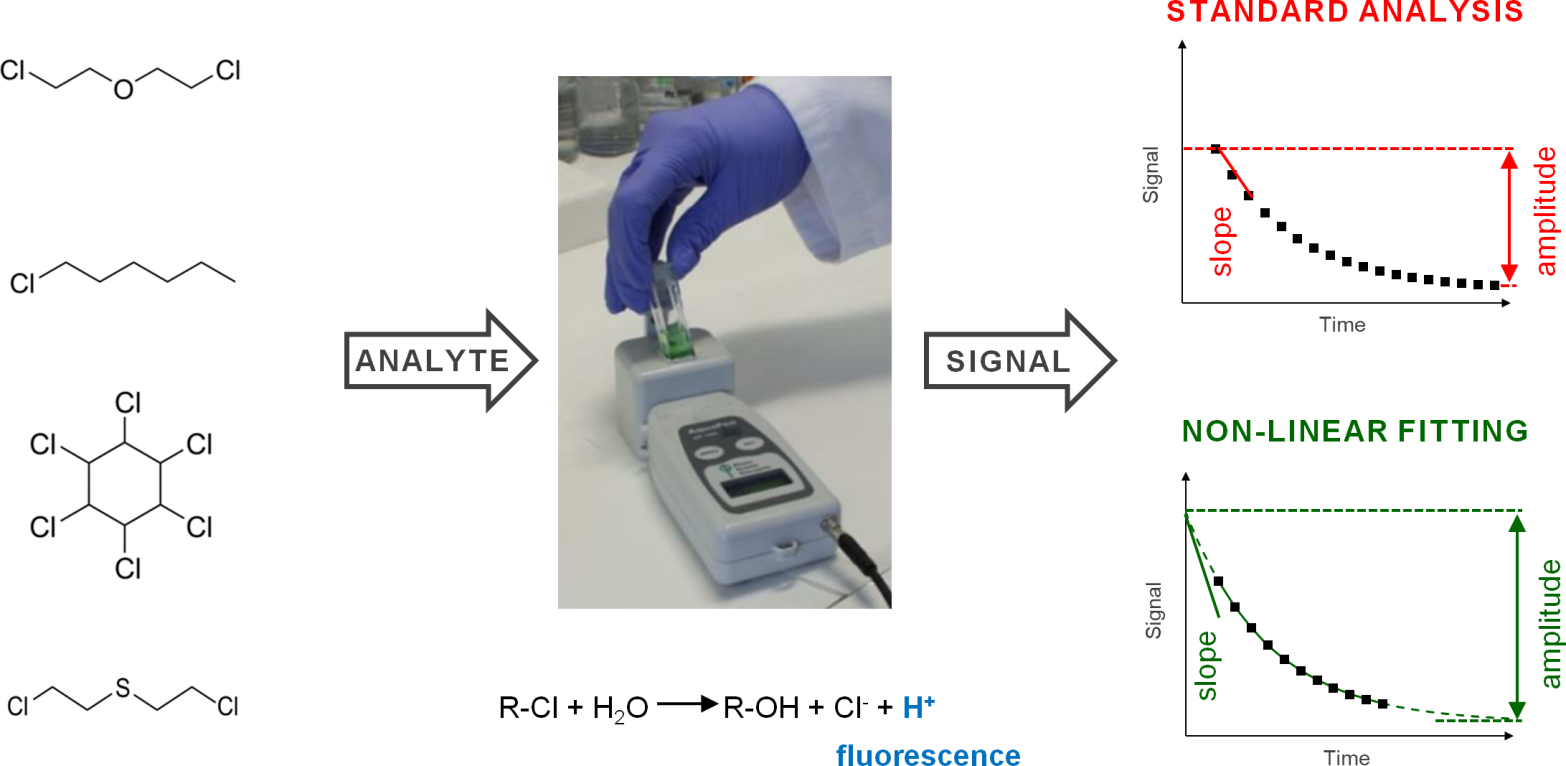
A schematic representation of the biodetection systems and data processing methods. Compounds to be detected by the developed biodevices are the surrogate bis(2-chloroethyl)ether, a common environmental pollutant 1-chlorohexane, a wide-spread toxic pesticide lindane (1,2,3,4,5,6-hexachlorocyclohexane, γ-hexachlorocyclohexane), and the chemical warfare sulfur mustard (left). The handheld fluorimeter and the model enzymatic reaction is given in the middle; in the case of the handheld fluorimeter, the reaction is initiated by adding the respective enzyme, and a fluorescent signal is recorded at ambient temperature and calculated as a ratio of the responses obtained after excitation at 485 and 410 nm, respectively. The two types of data analysis compared in this article are standard analysis mainly based on the raw data and a more sophisticated nonlinear curve fitting (right).

The paper is organized as follows. We will first define a standard analysis and nonlinear curve fitting, which are the two most intuitive ways of analyzing signals in enzyme kinetics. We then present a summary table highlighting pros and cons of those two approaches. After discussing each point separately, we will also compare the two types of the variables used for calibration, namely signal amplitudes and slopes. As model examples, we present two newly developed biosensing systems for detection of hazardous halogenated compounds (Fig 1) that consist of a pocket-size fluorimeter and enzymes haloalkane dehalogenase (EC 3.8.1.5) or γ-hexachlorocyclohexane dehydrochlorinase (EC 4.5.1.B1). The signal recorded by the device was processed using the abovementioned standard procedure and nonlinear curve fitting, and resulting calibration curves provided by those methods were compared and contrasted.

## Materials and methods

### Materials

The haloalkane dehalogenase LinB and γ-hexachlorocyclohexane dehydrochlorinase LinA from *Sphingobium japonicum* UT26 [23] were heterologously expressed in *Escherichia coli* BL21 and purified using metal affinity chromatography [24]. Biophysical characterization of the enzymes was reported earlier [25]. The ion-paired fluorescent pH indicator (HPTS-IP) was synthesized according to a previously described procedure [26]. Ampicillin sodium salt and isopropyl-β-D-thiogalactopyranoside were obtained from Duchefa (the Netherlands). Ethanol and methanol were purchased from Chromservis (Czech Republic). All other chemicals were purchased from Sigma-Aldrich (USA). All reagents were of analytical grade and used without purification. Solutions were prepared with deionized water with a resistivity of 18.2 MΩ cm using a Millipore Milli-Q water purification system (Millipore Inc., USA). Disposable plastic cuvettes having an optical length of 10 mm with lids were obtained from P-lab (Czech Republic).

### Instrumentation

A cuvette version of the AquaPen fluorimeter SN-AP-288 (Photon Systems Instruments, Czech Republic) was constructed using the solid-state LED containing a 410 and 485 nm band-limiting filter as a light source and a PIN photodiode with a 520 nm band-limiting filter as a detector. All the fluorescence measurements were performed at the angle of 90^o^. The device was connected to a computer via USB and the output signal was recorded using the FluorPen 1.0 program (Photon Systems Instruments, Czech Republic). The full relaxation of the enzyme-based biodevices was achieved within fifteen minutes. However, reliable slope estimates were obtained within the first three minutes. Curve-fitting was able to reduce the time necessary to estimate the relaxation amplitudes to six minutes by extrapolation (see Results and Discussion).

### Sample preparation and measurement procedure

The reaction mixtures (1 ml) in the cuvettes contained haloalkane dehalogenase LinB and dehydrochlorinase LinA, the pH indicator HPTS-IP at a concentration of 3 mM unless stated otherwise, 1 mM HEPES buffer (pH 9.0), and a halogenated substrate: the analog of warfare chemical sulfur mustard bis(2-chloroethyl) ether, the environmental pollutant 1-chlorohexane, and the pesticide 1,2,3,4,5,6-hexachlorocyclohexane. The reaction was initiated by adding the respective enzyme. The fluorescent signal was recorded at 23°C and calculated as a ratio of the responses obtained after excitation at 485 and 410 nm, respectively.

The concentrations of bis(2-chloroethyl) ether were determined using a 7890A gas chromatograph (Agilent Technologies, USA) with a DB-FFAP 30m × 0.25mm × 0.25μm capillary column (Phenomenex, USA) and a flame ionization detector. The temperature program began with an isothermal period at 40°C for 1 min, followed by a temperature increase to 170°C at a rate of 20°C per min. The concentrations of 1-chlorohexane and 1,2,3,4,5,6-hexachlorocyclohexane were quantified using the same instrument equipped with a ZB-5 30m × 0.25mm × 0.25μm capillary column (Phenomenex, USA) and an Agilent 5975C mass spectrometer (Agilent Technologies, USA). The temperature program began with an isothermal period at 40°C for 1 min, followed by a temperature increase to 250°C at a rate of 20°C per min and a final period of 2 min at 250°C.

### Data fitting

Curve fitting was performed by means of nonlinear least square minimization in MATLAB 2016a (The MathWorks, United States). Each progress curve was fitted independently according to analytical approximations to the Michaelis-Menten equations [27] and then standard errors were calculated based on the assumption of asymptotically normally distributed residuals (Wald statistics). Noise due to mixing was detected and accounted for using the following automated procedure. After the signal was obtained from the biodevice, it was subjected to two modeling procedures: (*i*) elimination of the noisy signal and (*ii*) curve fitting using the smooth segment of the signal to obtain reliable results for calibration curve construction. To identify the noisy segment of the signal, two timepoints were calculated for each curve: *t*_mixing_ (the starting point of mixing, i.e., the start of the noisy signal) and *t*_smooth_ (the starting point of the smooth observations, i.e., the end of the noisy signal). These timepoints were determined by iteratively performing linear fitting using data for 20 sequential data points, and computing the slopes of the fitted lines. The time *t*_mixing_ was defined as the final time point of the first segment for which the absolute value of the slope exceeded the threshold value of 0.2. The time *t*_smooth_ was defined as the starting point of the first 20-timepoint segment in the first sequence of ten iterative calculations over which the change in the calculated slope was less than 0.2.

Curve fitting to the signal was performed starting from *t*_smooth_ using a representation of the signal as a linear function of the product:

$${Signal}_{t}=AP_{t}+B,P_{t}=1-K_{M}W\left(\frac{1}{K_{M}}e^{\frac{1-V_{max}t}{K_{M}}}\right)$$

Here *W*(*x*) is a Lambert function [27] so that *P*_t_ is the solution to a normalized Michaelis-Menten kinetic equation for time *t*. The amplitude is given by the parameter *A*, and the initial slope is obtained by taking the first derivative at t = 0, which is equal to *A*.*V*_max_/(*K*_m_+1). *B* is a parameter responsible for the signal shift; *V*_*max*_ and *K*_M_ are constants from Michaelis-Menten framework. In the latter formula, the substrate concentration is fixed (normalized) at 1 since otherwise it overparameterized the system together with the amplitude factor *A*. For cases with low *K*_M_ values (Amplitudes vs. slopes section), one more exponential with parameters *C* and *D* was added:

$${Signal}_{t}=AP_{t}+B+C(1-e^{-Dt})$$

It should be noted that this work mainly focuses on describing some basic mathematical data treatments rather than seeking to discuss and evaluate every available function for curve fitting in specific situations. Excellent comprehensive reviews of curve fitting functions can be read elsewhere [28, 29].

## Results and discussion

Two biosensing systems for the detection of selected hazardous halogenated compounds have been developed and used for the validation of proposed approach (Fig 1 and Figure A in S1 File). In both cases, the enzymatic conversion of the analyte involves the proton release [30, 24]. These protons, in turn, affect the fluorescence of the ion-paired pH indicator HPTS-IP producing a biodevice signal which depends on the analyte concentration. The signal recorded by the device was processed using the standard analyses and nonlinear curve fitting, and resulting calibration curves and operational precision provided by those methods were compared. The standard analyses included: (*i*) amplitudes calculated as the difference between the maximal and minimal values of the signal in the chosen time window, and (*ii*) signal slopes determined by linear regression from the first few points of measurement. The curve fitting produced: (*i*) fitted amplitudes calculated using the exact formula from fitting; and (*ii*) fitted slopes calculated as the first derivative of the previous formula with respect to time, when the time is equal to zero. Table 1 summarizes the advantages and disadvantages of curve fitting in comparison to the standard analysis of biodevice signals with respect to the four key criteria that are discussed in more detail below: (*i*) simplicity of implementation on biodevices, (*ii*) the automatization of the signal collection and pre-treatment, (*iii*) the standard errors that one would expect to see based on repeated analysis of the same data sets during calibration, (*iv*) the ability to extrapolate signals to initial point of observation, e.g., to determine the sample pH, as well as to future times to reduce the detection time window.

**Table 1:** Comparison of different approaches to signal processing and calibration.

	Standard analysis	Nonlinear curve fitting
Amplitude estimates	**+**	**++**
Slope estimates	**+**	**++**
Simplicity of implementation	**++**	**+**
Automated noise handling	**-**	**++**
Calibration error mitigation	**-**	**++**
Extrapolation to starting or ending points	**-**	**++**
Reduction of measurement time	**+**	**++**

- not supported

+ low efficiency

++ high efficiency.

Standard calibration variables are total amplitudes of signals or their initial slopes quantifying initial velocities. Despite simple calculation, they are based on a limited amount of information from experimental data as their calculation excludes most of the measured points except for only a few. The results are affected by initial dead time of measurements, e.g., due to mixing, and often by reading from nonequilibrated data in the given timeframes. On the contrary, such incomplete data could be effectively analyzed by curve fitting and initial as well as equilibrium conditions can be extrapolated with high precision and provide more realistic estimates of total amplitudes or real initial rates. Moreover, automated data treatment can cut out the initial noise which can significantly augment the quality of the data for analysis. We will elaborate on each of those points below based on the datasets from the model experimental assays with a pocket-size fluorimeter. Although quantitative descriptions are obviously dependent on the assay under consideration, we believe that general principles of mathematical analyses are applicable to a wide range of analogous enzymatic biodevices.

### Simplicity of implementation

The first criterion–simplicity–is relatively straightforward: the standard analysis does not require any modeling or numerical fitting (explicit expressions exist for the linear regressions involved in the estimation of the slopes [29]). On the contrary, the nonlinear curve fitting requires determination of a model and algorithms for fitting. These algorithms, in turn, necessitate appropriate model progress curves, a program for fitting and parameter estimation, and hardware for program execution in biodevices. Nonetheless, progress curves have been studied extensively [28], fitting software is widely available, and many implementations tailored to biochemical applications exist [31]. It is also generally not problematic to incorporate some signal treatment units into biodevices [6]. We thus consider this disadvantage to be relatively insignificant. And in the case of initial slopes, the simplicity due to the lack of the model for fitting may often backfire: the initial few points might not align well leading to erroneous initial slope estimates based on a simple linear fit. In such cases either the smaller number of points has to be selected resulting in larger errors or nonlinear fit should be preferred.

### Automated noise handling

**Fig 2:**
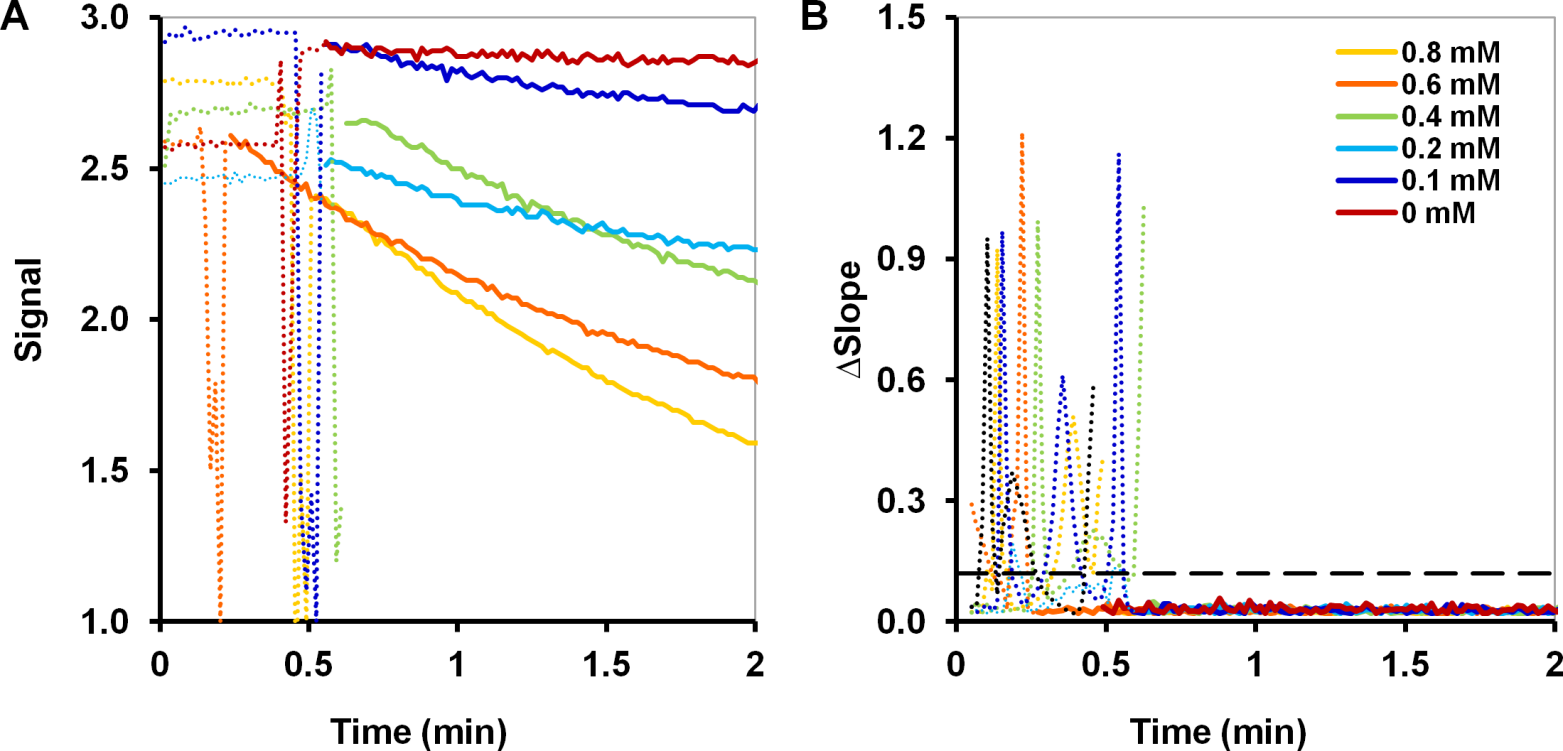
An example of raw data from measurements and the demonstration of the principle behind the automated selection of the relevant signal part for further analysis. (A) The signals with a substantial mixing noise in experiments using 2 mg of haloalkane dehalogenase LinB and different concentrations of bis(2-chloroethyl) ether; (B) The corresponding absolute values of the slope differences calculated by linear regression with a moving window; the solid lines correspond to the smooth signal region identified automatically by the software; a dashed line is the threshold for the definition of the smooth segment. A mixing period (dots) is usually the time from the beginning of an injection to a completely dissolved/mixed sample and, thus, this period varies in time among different measurements. The y-axes were cut to focus on the meaningful signal.

The simplicity of the standard analysis comes at a cost: it does not readily accommodate automated noise reduction. Raw experimental signals are typically not very smooth because there is usually at least one noisy segment corresponding to the mixing period (Fig 2A). Mixing of a substrate with enzyme or immersion of a transducer with an immobilized enzyme into a sample often lead to disruption of the signal and are considerably personalized. Therefore, signal pre-treatment is required to obtain a “smooth” dataset for the calculation of any of the four variables considered in this work. If the biodevice incorporates a data treatment unit, simple modeling can be used to automatically detect the smooth segment of the progress curve. If there is no such module, either manual intervention is required during every measurement or a fixed threshold time must be defined, which could lead to the loss of smooth signal if the threshold is too late or the inclusion of mixing noise if it is too early. Moreover, the dependence of the mixing on a person performing experiments further favors automated data treatment, which eliminates precisely mixing periods from analysis irrespective of how long and “noisy” they are.

In our system, we chose to calculate slopes over moving windows of time-points to enable discrimination between the noisy signal acquired during mixing when the slope varies strongly, and the smooth signal acquired later on when the calculated change in slope from segment to segment falls below some predefined threshold (Fig 2B). This enabled automatic identification of the smooth segment of each curve for further analysis. For example, for the sample with the substrate concentration of 0.6 mM the smooth signal started 26 seconds after the initiation of the sensor, whereas for 0.4 mM it was 57 seconds. Hence, manual editing would have either excluded 30 seconds of the meaningful signal for 0.6 mM or included 30 seconds of the noisy signal for 0.4 mM concentrations. And a close comparison of the signal segments automatically chosen by the software (solid lines in Fig 2B) with the actual signal and noise revealed that this relatively simple noise handling procedure identified the smooth regions in the signal with a precision of a few seconds.

### Calibration error mitigation

**Fig 3:**
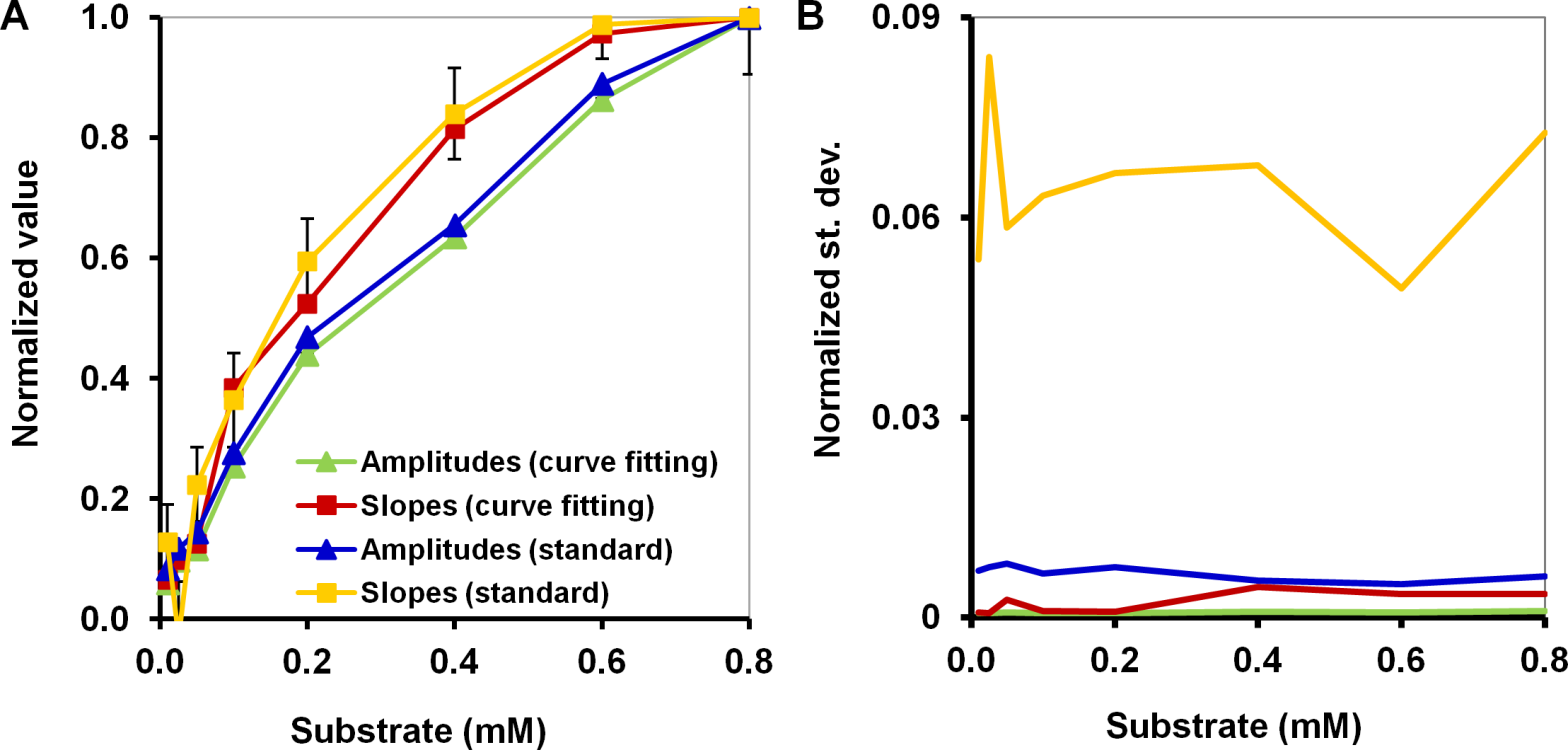
Comparison of the calibration curves obtained via the two approaches: The standard analysis and nonlinear curve fitting. (A) Calibration curves for the biodevice based on experiments with 0.8 mg of haloalkane dehalogenase LinB and sulfur mustard surrogate bis(2-chloroethyl) ether; (B) The normalized standard deviations of the estimates of the corresponding values used for calibration; the average normalized standard deviations of estimates (bars) were 0.8, 2.2, 6.7, and 64.5 ·10^−3^ for amplitudes and slopes from nonlinear curve fitting, and amplitudes and slopes from the standard analysis, respectively.

In addition to the substantial noise that occurs at the start of the measurement period, artifacts such as sudden spikes or troughs can also appear in the middle of an otherwise smooth data set. This is why the standard error of the fit is one of the criteria used to evaluate the two approaches. The nonlinear curve fitting resulted in the smallest standard deviations for the estimates (Fig 3) of the amplitudes (the normalized average standard deviation was 0.8·10^−3^) and slopes (2.2·10^−3^). The standard analysis showed larger values for both variables (6.7·10^−3^ and 64.5·10^−3^ for amplitudes and slopes, respectively). The linear slopes in the standard analysis have significant standard errors even despite the linear fitting used because the slope is mainly determined by the few points at the beginning of the measurement. The amplitudes in the standard analysis are more robust in this respect, although they do depend on the points selected, which are also prone to error. In the curve fitting, the fitted slopes exhibited smaller standard errors than those from the standard analysis mainly because fitting enables the entire curve to contribute to the determination of the parameter. Quite expectedly, the smallest standard errors were observed for the fitted amplitudes.

It should also be noted that both amplitude variables yielded more linear calibration curves than did the slopes (the relative deviations from a straight line are 22%, 27% for fitted and signal amplitudes *versus* 43%, 48% for fitted and signal slopes, respectively, based on the area under the curves). In other words, the normalized calibration curves for the slope variables were more concave and exhibited faster saturation upon increases in substrate concentration, which is disadvantageous because any curvature reduces the sensitivity of the biodevice to the region where it occurs. For instance, a 20% deviation from the maximal signal in Fig 3A will produce ranges of approximately 0.6–0.8 mM for amplitudes but only 0.4–0.8 mM for slopes, making calibration based on amplitude variables more sensitive to substrate concentrations in that range. This is normally the case because the initial slopes are more prone to saturation than amplitudes, as in the case of Michaelis-Menten kinetics [32]. Ideally, a calibration curve should be a straight line (the zero-relative deviation of the area under curves) indicating uniform sensitivity to substrate concentrations in the range of interest, which is why in the case of initial velocities a Lineweaver–Burk reciprocal plot may be used to obtain straight lines [33]. However, this transformation complicates the calculation of standard errors as the corresponding transformation of the error term is quite non-trivial; therefore, the amplitude variables are better choices with respect to this criterion.

### Extrapolation to starting and ending points

**Fig 4:**
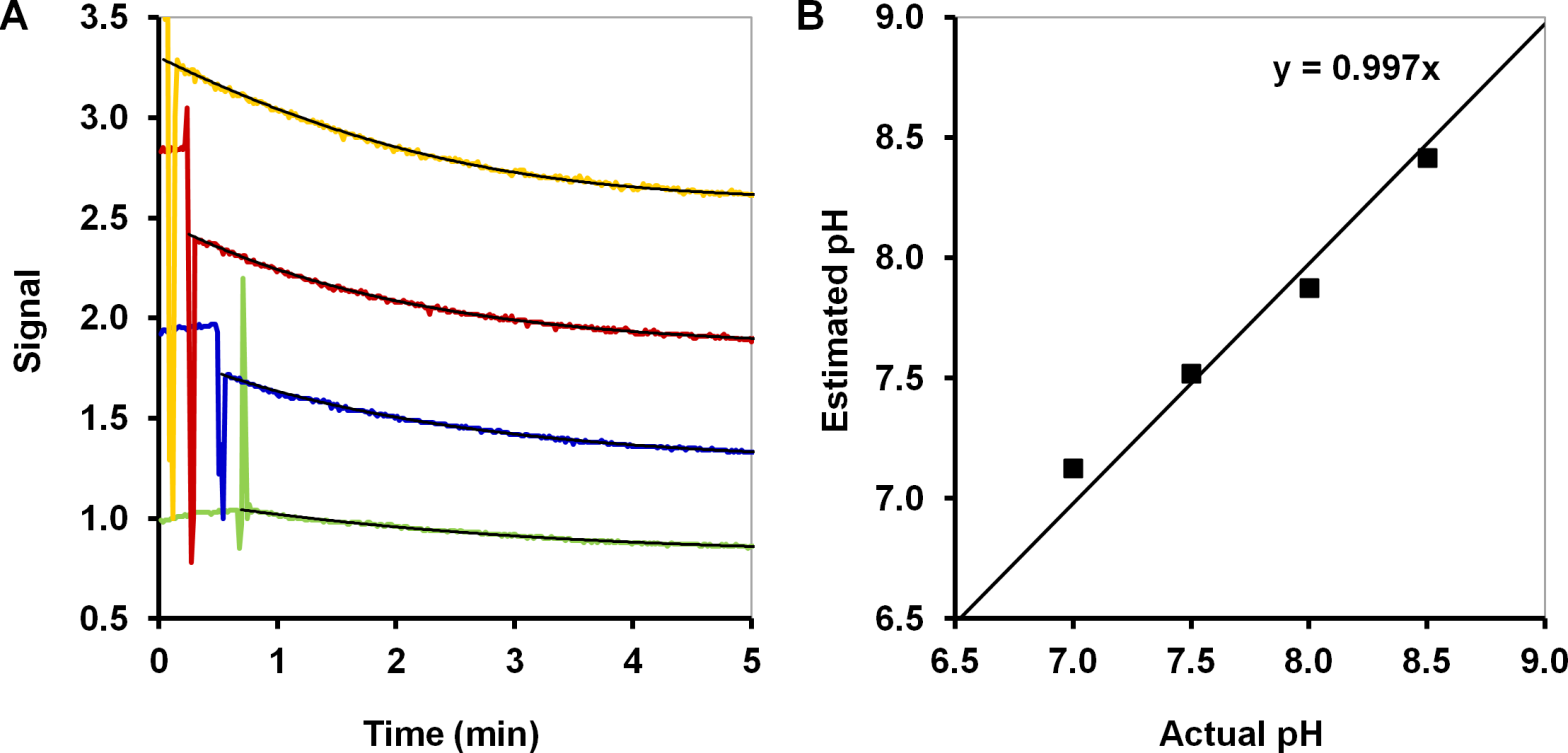
An example of the sample pH estimation by extrapolation of the fitted curve to the starting point of the measurements. (A) The response of the biodevice based on experiments using 0.8 mg of haloalkane dehalogenase LinB and 0.4 mM of sulfur mustard surrogate bis(2-chloroethyl) ether at four different pH values; (B) the estimated pH based on the initial point of the fitted curve.

The use of simple variables in the standard analysis also makes it impossible to extrapolate whereas in the nonlinear curve fitting extrapolation can be implemented straightforwardly. There are two applicable types of the extrapolation: the extrapolation to the initial time and the future time outside of the measurement window. One potential application of curve fitting and extrapolation to the initial state is in the determination of the sample pH (Fig 4), which may be useful when attempting to determine whether the pH is within the calibrated range. If it is not the case, the pH should be adjusted to increase the precision of the measurement or another part of the calibration surface should (automatically) be selected given there exists such a pH dimension of calibration.

**Fig 5:**
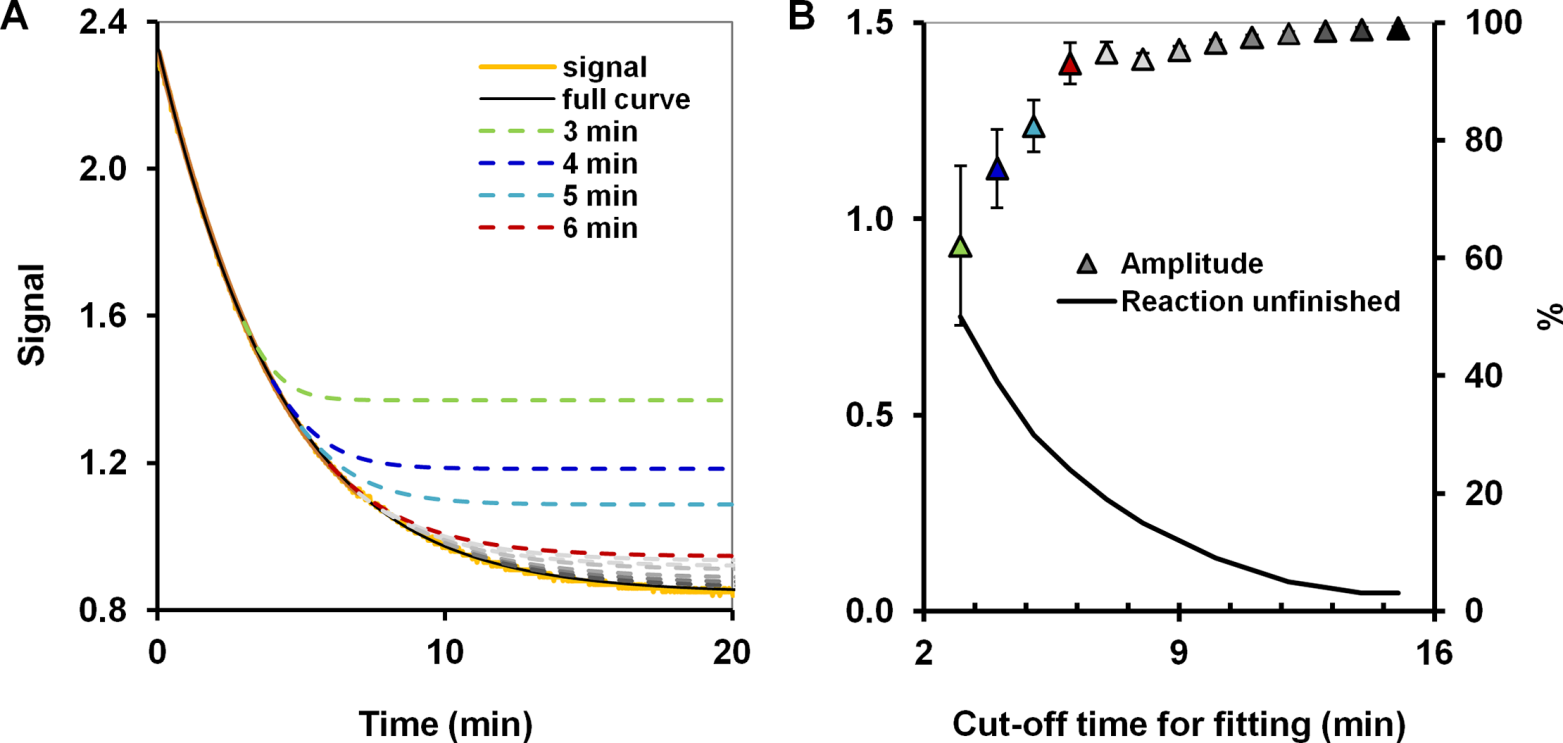
Extrapolation of the curves fitted based on the different time windows to the equilibrated signal at later times in the nonlinear curve fitting. (A) A signal based on experiments with 0.8 mg of haloalkane dehalogenase LinB and sulfur mustard surrogate bis(2-chloroethyl) ether and the (extrapolated) nonlinear fit for different cut-off times (in the legend); (B) The values of the amplitude as a function of the cut-off time for fitting (left), and the proportion of the curve excluded from the fitting expressed as a percentage (right).

The extrapolation to the future times may significantly reduce the time required for analysis. Indeed, in case of amplitudes in contrast to the standard analysis, where the whole relaxation curve must be obtained to provide reliable measures of signal amplitudes, in the nonlinear curve fitting a partial signal might already be enough to estimate the amplitudes with sufficient precision. In Fig 5 we investigated a trade-off between a time window for measurement and an acceptable level of data quality, i.e., the error of extrapolation to infinity. We tested the effect of limiting the timeframe used for curve fitting from the first 3 minutes to the first 15 minutes. Observation windows as short as 6 minutes proved to be adequate to obtain amplitude values very similar to those derived by considering complete signal curves, and the confidence intervals for amplitudes determined using these shortened windows were very similar to those based on complete curves. It might be expedient to note at this point again that this extrapolation is also only possible because of the model available, i.e., if the nonlinear curve fitting was used; the standard analysis is incapable of providing such information.

### Amplitudes vs. slopes

As was pointed out earlier, substrate concentration usually affects both signal amplitudes and slopes. Based on enzyme kinetics, assuming the pH change is within a linear segment of the calibration curve for the dye, the amplitude is linearly proportional to the total substrate concentration. In contrast, even in the simplest Michaelis-Menten setting, the initial slope is a linear function of the initial reaction rate, which is proportional to the substrate concentration but in a hyperbolic manner, i.e., *V*_max_ [S] / (*K*_M_+[S]). Consequently, while both absolute values of amplitudes and slopes increase with the increase of the substrate concentration, the dependence is different. For instance, unlike amplitudes, slopes can be computed without obtaining a full signal curve, and any reduction of the detection time is usually preferred other things being equal. However, as was demonstrated in the previous section, amplitudes from the nonlinear curve fitting may also provide reasonable timeframes for quick sample analysis since they may be explicitly derived by extrapolation of the equations fitted in a short time window to the infinite time. Moreover, in the case of low *K*_M_ values, the enzyme becomes saturated, which results in the relatively constant initial reaction rate (close to *V*_max_) and unlike amplitude, the slope becomes insensitive to the substrate concentration. When comparing slopes and amplitudes, the latter are more robust to the occurrence of more complex than the simple Michaelis-Menten kinetics in the signal curves.

**Fig 6:**
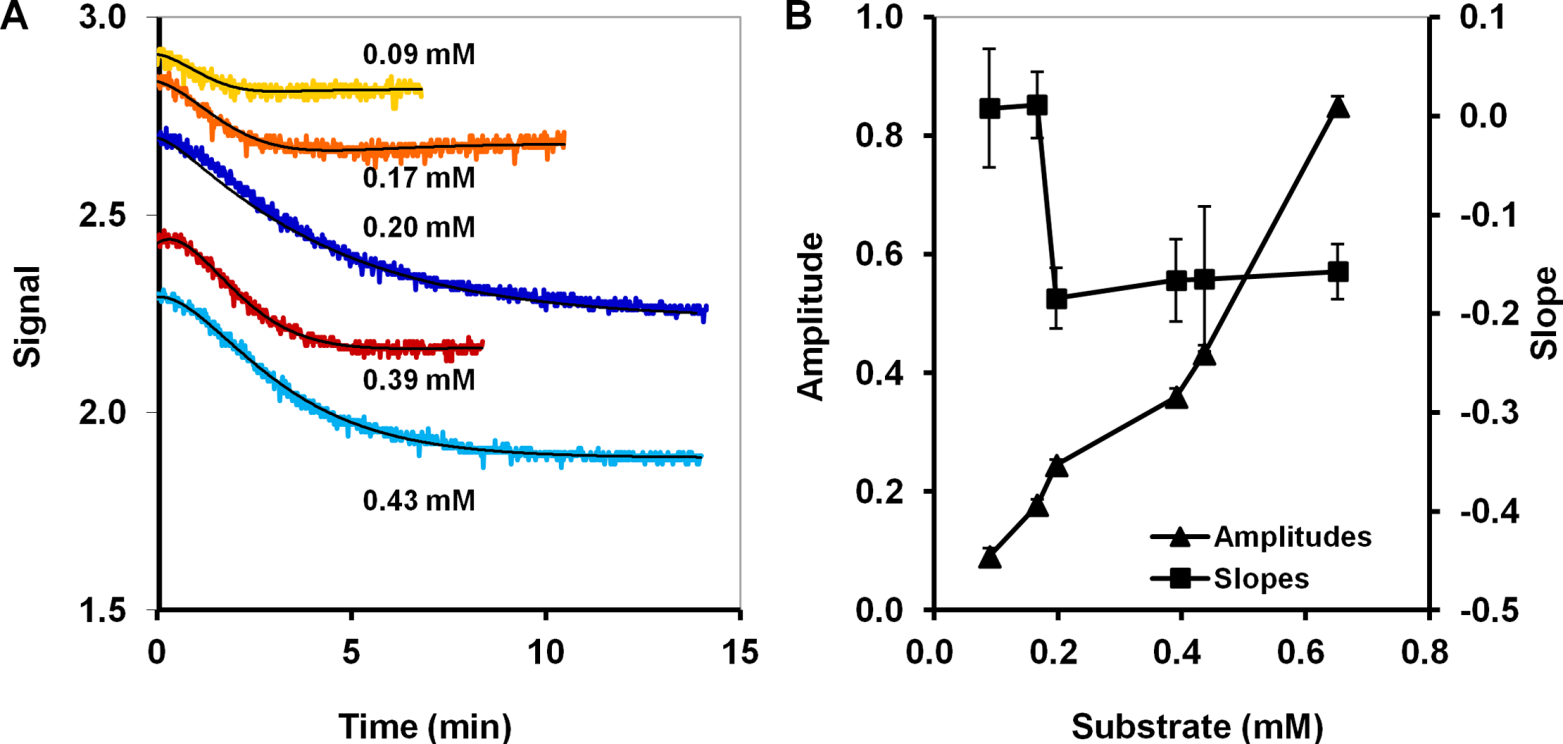
Poor calibration based on slope versus a better quality calibration based on amplitudes in the case of a complex reaction kinetics. (A) The response of a biodevice based on experiments using 0.8 mg of haloalkane dehalogenase LinB and different concentrations of environmental pollutant 1-chlorohexane; (B) calibration points for slopes and amplitudes.

This is illustrated by an example observed in experiments with LinB and environmental pollutant 1-chlorohexane (Fig 6). LinB has a low *K*_m_ value of 0.005 mM for this substrate [34]. The slopes for different substrate concentrations were expected to be very similar even when the substrate concentration was very low because most of the observations would be acquired in the flat region of the Michaelis-Menten curve. On the contrary, the measured and fitted amplitudes should still be proportional to the initial substrate concentrations because they are based on the absolute change in pH caused by substrate conversion but not the kinetics to reach this equilibrium parameter. However, in our experiments complex kinetics were observed: the signal curve exhibited two distinct phases, the first of which exhibited a relatively constant initial slope but was too brief to permit separate parameter estimation for both phases. Consequently, slope-based calibrations obtained by considering complete signal curves lacked robustness and exhibited poor fit qualities; these problems were not observed for calibrations based on amplitudes.

**Fig 7:**
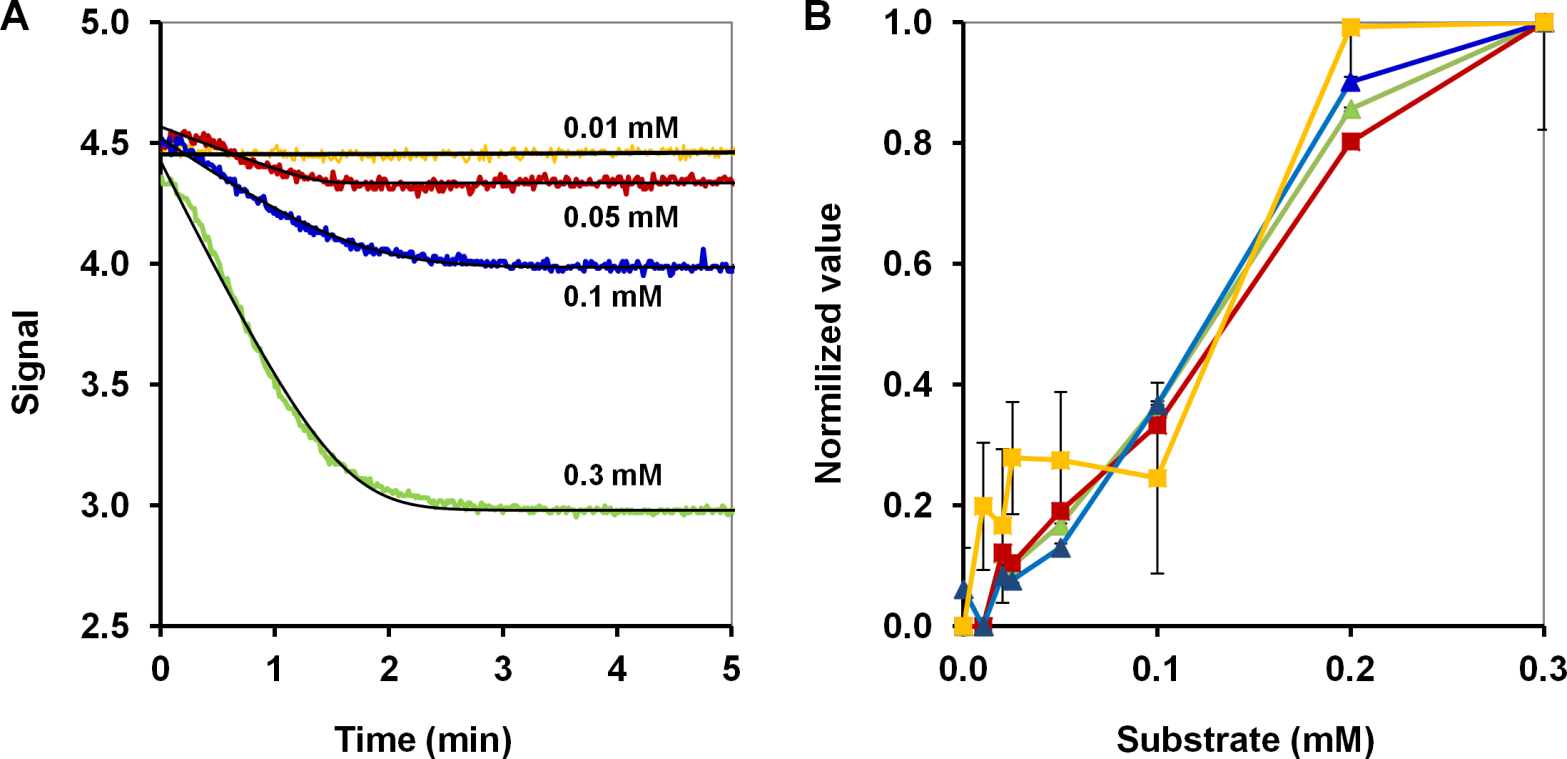
Comparison of the four candidate variables for calibration of a biodevice for the detection of lindane. (A) The response of a biodevice based on experiments with 0.1 mg of dehydrochlorinase LinA and different concentrations of environmental pollutant lindane and the corresponding fitted curves (black); (B) Calibration curves for the biodevice: amplitudes from the nonlinear curve fitting (green), slopes from the nonlinear curve fitting (red), amplitudes from the standard analysis (blue), and slopes from the standard analysis (yellow); the average normalized standard deviations of estimates (bars) were 2, 6 ·10^−3^ for the amplitudes and slopes from nonlinear curve fitting and 7, 126 ·10^−3^ for the amplitudes and slopes from the standard analysis, respectively.

The above-mentioned case is quite extreme, and the slope estimates do not have to be as poor as in Fig 6 for more favourable kinetic constants, but if there are some delays in the development of signals, the curve fitting will provide more reliable estimates of the slopes. For instance, apart from the biodevice for detection of chemical warfare, we assembled a similar biodevice for detection of hazardous pesticide lindane, listed by the Stockholm Convention on Persistent Organic Pollutants. The importance of such a biodevice is difficult to overestimate: 1,2,3,4,5,6-cyclohexachlorohexane (HCH) production in Sabinanigo between years 1975 and 1988 alone resulted in approximately 115,000 tonnes of hazardous organochlorine wastes, largely HCH waste isomers [35]. As can be seen from the calibration curves (Fig 7), the signal develops rather slowly upon mixing so the initial slopes from the standard analysis of the signals are again of little value for calibration. Nonetheless, fitted slopes demonstrated the quality of fit comparable with that of amplitudes.

**Fig 8:**
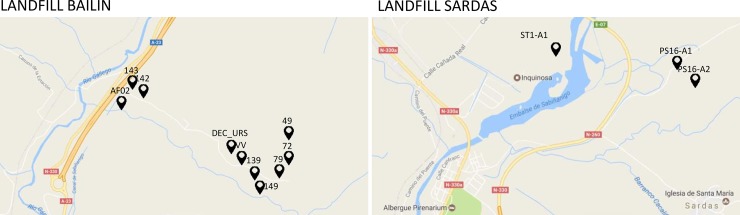
The 13 sites selected for testing the detection of lindane in Sabinanigo.

Based on the amplitudes from the fitting, the acquisition system was also tested on real samples taken from thirteen places nearby the waste sites in Sabinanigo (Fig 8), which was done in collaboration with SARGA, Zaragoza (Spain). The results were also confirmed using standard GC. In three different localities, signals obtained with the biosensor resulted in a detectable concentration, albeit at the lower end of the detection range. For the remaining ten sites, the concentration confirmed by GC was below the detectable levels for the biosensor of 2.9 mg/L (Table A in S1 File). These results demonstrate both the applicability of the biosensor in field measurements as well as (dis)advantages of the acquisition system compared to the standard analytical technique, i.e., the portability and ease of use of the former versus more precision and higher sensitivity of the latter.

It is worth mentioning, that the advantages of the nonlinear curve-fitting with fitted initial slopes and amplitudes are not limited to the acquisition systems discussed in the current study. They can also be observed in other enzyme-based biodevices provided they capture relaxation signals, i.e., record signal versus time. Bidmanova and co-workers [24] describe another portable hand-held biosensor used at contaminated sites in Pančevo, Serbia, where a chemical factory was bombarded during the Yugoslav wars in 1999. This biosensor EnviroPen (Photon Systems Instruments, Czech Republic) is based on haloalkane dehalogenase DhlA and a fluorescence pH indicator 5(6)-carboxynaphtofluorescein. The biosensor produced relaxation curves with similar features to those described here. Thus, if an enzyme-based biosensor produces time-dependent signals, it may benefit from a more advanced curve-fitting signal processing. This approach leads to automatic noise removal, more robust parameter estimation, and the possibility of extrapolation to the initial time or equilibrium.

## Conclusions

The aim of this study was to rigorously compare different methods of signal analysis and demonstrate the advantages of using mathematical data treatment techniques to improve the performance of newly developed bioanalytical devices. Models derived from raw signal allow more precise quantification of measurements generated during biodevice development than the information obtained by standard techniques. In particular, we demonstrated how simple signal processing helps to optimize biodevices for the detection of various hazardous chemicals: the analog of warfare chemical sulfur mustard bis(2-chloroethyl) ether, the common environmental pollutant 1-chlorohexane, and a toxic pesticide 1,2,3,4,5,6-hexachlorocyclohexane and related molecules, listed by the Stockholm Convention on Persistent Organic Pollutants. The tested concentrations revealed positive correlation for signal variables in the range of 0.01–0.8 mM for bis(2-chloroethyl) ether and in the range of 0.01–0.3 mM for γ-hexachlorocyclohexane. As far as applications of the developed devices are concerned, the nonlinear curve fitting and data analysis enable automated treatment of the initial noise generated during quite personalized sample mixing, reducing the measurements’ dependence on the mixing time and the need for manual signal processing. It also enables reliable extrapolation to initial conditions, making it possible to precisely quantify actual sample characteristics. In the case when only partial relaxation was measured, the nonlinear curve fitting allows extrapolation to the equilibrium, reducing the necessary detection time window even when the amplitudes are used. After comparison of the calibration based on amplitudes and slopes determined by the fitting to that from the standard analysis, we conclude that amplitudes from curve fitting should be preferred in most cases because of their robustness and powerfulness. We expect that presented advanced signal processing will find a broad use in the development and operation of new biodevices.

## Supporting information

S1 File Optimization of the biodevices, stability analysis, and field measurement results in Sabinanigo. (PDF)
