# Supplementary material for: Sensitive operation of enzyme-based biodevices by advanced signal processing
Source: PLoS One. 2018 Jun 18;13(6):e0198913. doi: 10.1371/journal.pone.0198913 (PMC6005535; doi:10.1371/journal.pone.0198913)
Supplement: S1 File — (PDF) [file pone.0198913.s001.pdf]

Supplement for Sensitive operation of enzyme-based biodevices by advanced signal processing

S. Mazurenko, S. Bidmanova, M. Kotlanova, J. Damborsky, Z. Prokop

The optimization of biodevices typically involves selecting optimal concentrations of system components and optimizing with respect to other factors that affect analytical performance such as the pH and ionic strength.

Optimization of enzyme concentrations

During the development of a biosensor one must choose an optimal enzyme concentration. In our system, we discovered that the time of conversion is almost insensitive to the substrate concentration in the tested range (Fig Aa-b). Therefore, the average time required for complete conversion is an intuitively appealing metric to use when attempting to determine an optimal enzyme concentration. The modeling substantially enhanced this quantification of the data. The time at which 95% of the signal had appeared was defined as the conversion time, and an explicit equation for the signal curve $y=f(t,b)$ was selected and fitted using non-linear least squares minimization to obtain the optimal set of parameters b^*:

$$f(t,b) = b_1+b_2 \bullet (1 - b_3 \bullet W(1 / b_3 \bullet \exp\{(1 - b_4 \bullet t) / b_3\})).$$

Here t stands for time and b is the vector of parameters and $W(x)$ is the Lambert function (see Materials and Methods for more information). Then the relaxation time for each curve t_{relax} was calculated using the equation $f(t_{relax},b^*)=0.95\cdot(y_{max}-y_{min})$. For simple exponential functions, the solution to the previous equation can be found analytically. However, when using the Michaelis-Menten approximation, a rapid numerical solution was obtained (Fig Ab). Surprisingly enough, without modeling, the relaxation time cannot be readily estimated from the dataset of points; visual inspection suggests that different substrate concentrations (Fig Aa) should have completely different relaxation times, in contrast to the similar times obtained by the curve fitting (Fig Ab). In our model example, a 15 min measurement window appeared to be reasonable, so 0.8 mg of LinB was selected as the optimal value.

Optimization of dye concentrations

The effect of varying the dye concentration is two-fold: increasing the dye concentration would be expected to increase the signal amplitude up to a certain point, beyond which no significant improvement would occur or, worse, the excess of the dye may start to adversely affect the signal. In our model bioassay, simple sigmoid fitting of the amplitudes for different dye concentrations revealed that a dye concentration of 18 µM yielded an amplitude equal to 96% of the maximum observed value (Fig Ac-d). Consequently, that concentration of the dye was selected for general use.

Optimization of pH

Usually, the region of the steepest slope is preferred because in that region small changes in pH will produce large changes in the signal. Although only one starting pH value is usually selected, the choice of this point has to account for the change in pH as the reaction progresses. It is therefore necessary to determine an optimal range rather than a single optimal value. Once the non-linear curve fitting was performed and a model equation was derived, the first derivative was taken analytically to determine both the most sensitive

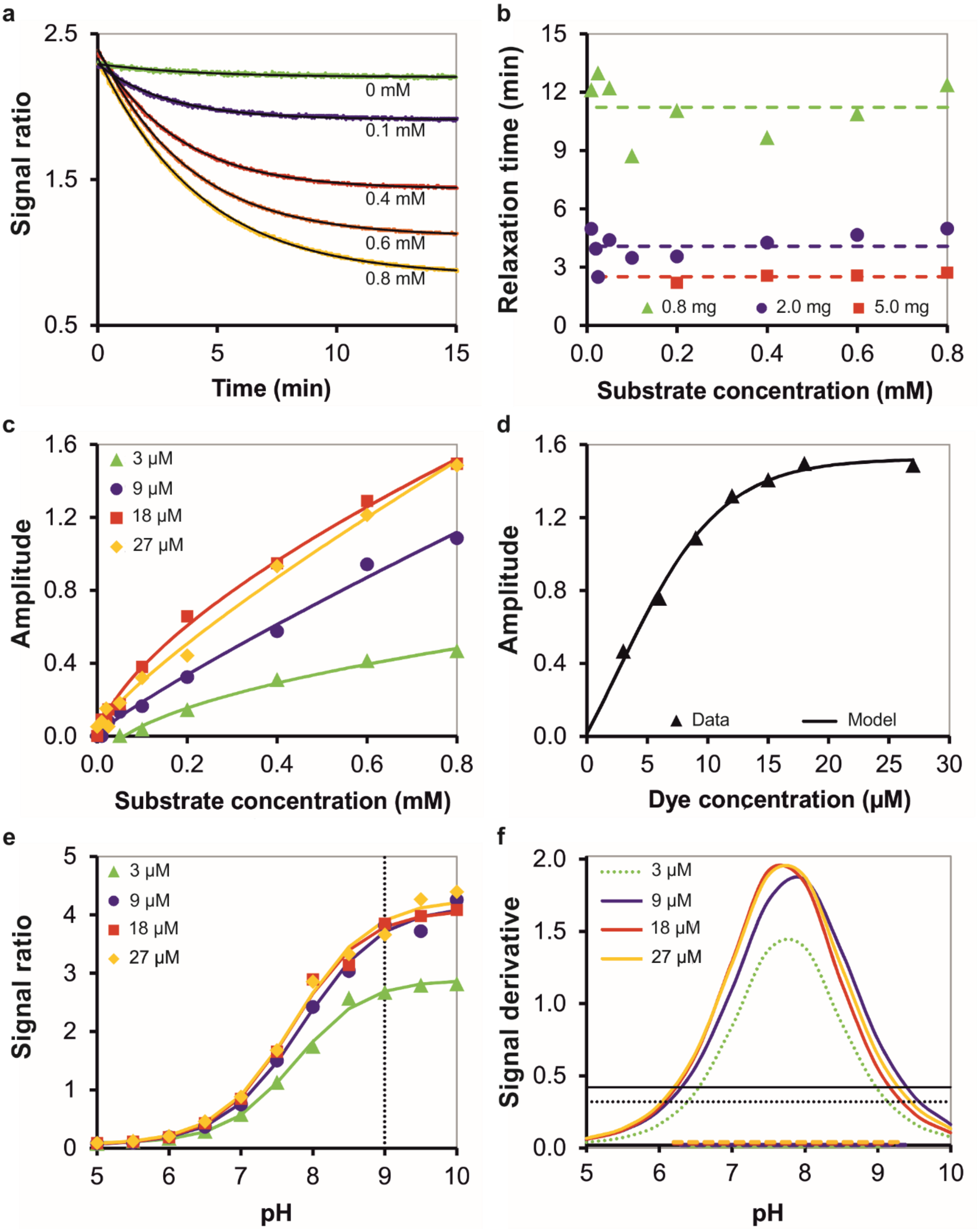

Fig A. The optimization of biodevices. (a) The response of a model bioassay based on 0.8 mg of haloalkane dehalogenase LinB and different concentrations of bis(2-chloroethyl) ether; (b) Relaxation times corresponding to 95% conversion for 0.8 mg, 2.0 mg, and 5.0 mg of the enzyme, with the average relaxation times in each case indicated by the dashed lines; (c) Calibration curves for the bioassay based on 0.8 mg of haloalkane dehalogenase LinB and bis(2-chloroethyl) ether with different concentrations of the fluorescent dye HPTS-IP; (d) The fitted amplitudes for experiments with 0.8 mM of the substrate and different dye concentrations; (e-f) The response of the bioassay as a function of pH with fitted curves (e), and the first derivatives of the corresponding curves (f) with the most sensitive region highlighted. Different concentrations of the fluorescent dye HPTS-IP were tested.

point, which is the peak of the first derivative, and the most sensitive region, which is defined as the area of the peak above a certain threshold (Fig Af). A sigmoid function was used for each pH profile curve:

$$f(x,b) = b_1 + (b_2 - b_1) / (1 + \exp\{(x - b_3) / b_4\}).$$

Its derivative is then given by

$$f'(x,b) = -1 / b_4 \bullet \exp\{(x - b_3) / b_4\} \bullet (b_2 - b_1)/(1 + \exp\{(x - b_3) / b_4\})^2.$$

In our model case, we selected a sensitivity threshold corresponding to 5% of the total signal change per pH unit because values below this level were barely distinguishable from noise. The pH range for which the derivative exceeded this threshold was 6.5 to 9.0. Since the pH decreases as the underlying reaction proceeds, the optimal starting pH was identified as 9.0.

Stability of biorecognition elements

The long-term stability of biocatalyst is a crucial parameter to be considered for the application of enzymes in industrial biocatalysis. While the main biophysical characterisation of the enzymes involved has already been published elsewhere[1], we additionally investigated the long-term storage stability of free haloalkane dehalogenase LinB from *Sphingobium japonicum* UT26[2] at 4 °C and 21 °C for the current study.

Haloalkane dehalogenase LinB was overexpressed in *Escherichia coli* BL21(DE3) containing plasmid pAQN::*linB-UT*. The His-tagged LinB was purified using the chromatographic column HR 16/10 (GE Healthcare, Sweden) with Ni-NTA sepharose (Quiagen, Germany) attached to the FPLC system ÄKTA (GE Healthcare, Sweden) as described earlier[3]. The prepared LinB was dialysed against 50 mM phosphate buffer, pH 7.5. Sodium azide was added to LinB to final concentration of 2 mM. The aliquots of LinB (250 µl, 1.4 $mg \cdot l^{-1}$) were pipetted into Eppendorf tubes and stored at 4 °C and 21 °C.

Enzymatic activity was determined in 10 ml of 100 mM glycine buffer (pH 8.6) at 37 °C using Iwasaki method[4]. The substrate 1,2-dibromoethane was added to create a final concentration of 8.7 mM, determined by gas chromatograph (Trace GC, ThermoQuest Scientific, Great Britain). The reaction was started by addition of LinB to the reaction mixture and terminated by mixing with 35% (v/v) nitric acid. Increasing concentrations of reaction products (bromide ions) were measured with mercuric thiocyanate and ferric ammonium sulphate at 460 nm by spectrophotometer Sunrise (Tecan, Switzerland). The amount of reaction products was determined from a calibration curve prepared using sodium bromide as a standard solution. The concentration of LinB in reaction mixture was determined by Bradford method[5]. The

---

[1] Koudelakova T., Bidmanova S., Dvorak P., Pavelka A., Chaloupkova R., Prokop Z., Damborsky J. (2013): Haloalkane dehalogenases: biotechnological applications. *Biotechnol. J.* 8: 32-45.

[2] Nagata Y., Nariya T., Ohtomo R., Fukuda M., Yano K., Takagi M. (1993): Cloning and sequencing of a dehalogenase gene encoding an enzyme with hydrolase activity involved in the degradation of hexachlorocyclohexane in *Pseudomonas paucimobilis*. *J. Bacteriol.* 175: 6403-6410.

[3] Nagata Y., Hynkova K., Damborsky J., Takagi M. (1999): Construction and characterization of histidine-tagged haloalkane dehalogenase (LinB) of a new substrate class from a γ-hexachlorocyclohexane-degrading bacterium, *Sphingomonas paucimobilis* UT26. *Protein Expr. Purif.* 17: 299-304.

[4] Iwasaki I., Utsumi S., Ozawa T. (1952): New colorimetric determination of chloride using mercuric thiocyanate and ferric ion. *Bull. Chem. Soc. Jap.* 25: 226.

[5] Bradford M.M. (1976): A rapid and sensitive method for the quantification of microgram quantities of protein utilizing the principle of protein-dye binding. *Anal. Biochem.*72: 248-254

enzymatic activity during storage was expressed as a relative activity in comparison to the initial activity of LinB.

The activities of haloalkane dehalogenase LinB stored at 4 °C and 21 °C were investigated for 12 months. Obtained results are summarized in Fig B. Enzymatic activity of haloalkane dehalogenase LinB observed during the storage at 4 °C and 21 °C for 12 months was comparable to the initial enzymatic activity. These data suggest that enzyme LinB stored at both temperatures is stable for more than one year.

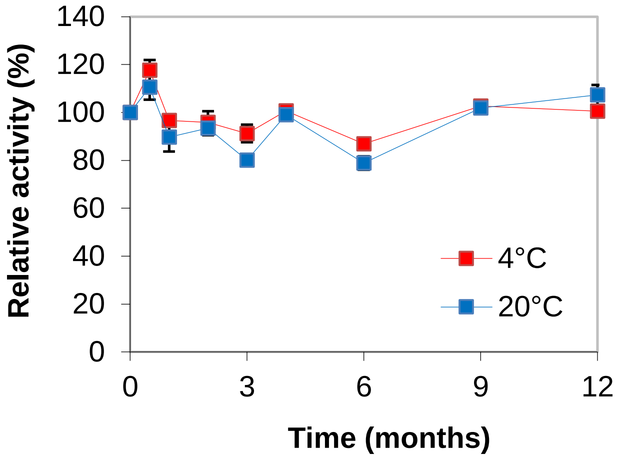

Figure B. Storage stability of haloalkane dehalogenase LinB. The stability was tested in 50 mM phosphate buffer (pH 7.5) with 2 mM sodium azide at 4 °C and 21 °C. Error bars show standard deviation from three replicated measurements.

Detection of HCH in Sabinanigo

Table A: The concentrations of HCH isomers measured at different sites in Sabinanigo. While the concentrations obtained by GC were at the low end of the calibration range of the biosensor, the concentrations above the detectable limit of 2.9 mg/L were correctly estimated based on the signal amplitudes.

Site	GPS coordinates	Signal amplitude	HCH concentration (biosensor, mg/L)	HCH concentration (GC, mg/L)
DEC_URS	42°29'08.3"N 0°21'35.4"W	0.15	4.5	3.7
VV	42°29'05.6"N 0°21'31.9"W	0	0	1.2
49	42°29'07.5"N 0°21'16.2"W	0	0	2.4
72	42°29'05.4"N 0°21'17.3"W	0.10	3.0	3.4
79	42°29'03.5"N 0°21'18.8"W	0	0	1.3
139	42°29'02.2"N 0°21'27.3"W	0.09	2.9	3.2
149	42°28'58.6"N 0°21'26.5"W	0	0	0.1
AF02	42°29'18.4"N 0°22'11.1"W	0	0	0
142	42°29'21.2"N 0°22'03.1"W	0	0	0.1
143	42°29'22.3"N 0°22'05.5"W	0	0	0
ST1-A1	42°30'56.6"N 0°20'58.8"W	0	0	0.2
PS16-A1	42°30'52.3"N 0°20'19.7"W	0	0	0.4
PS16-A2	42°30'49.3"N 0°20'14.9"W	0	0	1.0
